# Dynamics of social network emergence explain network evolution

**DOI:** 10.1038/s41598-020-78224-2

**Published:** 2020-12-14

**Authors:** Caleb Pomeroy, Robert M. Bond, Peter J. Mucha, Skyler J. Cranmer

**Affiliations:** 1 Department of Political Science, The Ohio State University, Columbus, OH 43210 USA; 2 School of Communication, The Ohio State University, Columbus, OH 43210 USA; 3 Carolina Center for Interdisciplinary Applied Mathematics, Department of Mathematics, University of North Carolina, Chapel Hill, NC 27599 USA

**Keywords:** Social evolution, Human behaviour, Complex networks

## Abstract

Networked systems emerge and subsequently evolve. Although several models describe the process of network evolution, researchers know far less about the initial process of network emergence. Here, we report temporal survey results of a real-world social network starting from its point of inception. We find that individuals’ ties undergo an initial cycle of rapid expansion and contraction. This process helps to explain the eventual interactions and working structure in the network (in this case, scientific collaboration). We propose a stylized concept and model of “churn” to describe the process of network emergence and stabilization. Our empirical and simulation results suggest that these network emergence dynamics may be instrumental for explaining network details, as well as behavioral outcomes at later time periods.

## Introduction

A host of social, biological, and technological systems emerge and subsequently evolve as networks^1–6^. Network models parsimoniously represent the evolutionary dynamics of these complex systems. Simple, lower-order processes that govern tie formation and dissolution, such as transitivity, reciprocity, fitness, homophily, and preferential attachment^7–10^, in turn generate important structural features like transitive triplets, motif distributions, and skewed degree distributions^11–13^. In human populations, these processes help to explain patterns of inequality, health outcomes, and cooperative stability^14–19^, among others.

The origins of social networks, however, remain elusive. New social environments, such as geographic relocation or the inceptions of new jobs and educational programs, give rise to nascent, previously unobserved social networks. In an ideal typical case, new social networks emerge when unfamiliar individuals enter a new situation and form relationships for the first time. For example, a university’s first-year orientation leads to the first interactions between most incoming students. Students’ social networks might emerge gradually according to well-known processes of constant growth, e.g. Ref. ^9^. Alternatively, initial interactions might differ from later evolutionary periods marked by more stable relationships, in which case tie formation behavior varies across time.

Here, we investigate the latter network emergence case, which poses a greater challenge to extant evolutionary models and theory. We propose that a broad cross-section of emerging networks undergo a process of “churn,” marked by an expansion and contraction of ties. As networks emerge, individuals initially form several previously unobserved ties. Many of these initial ties eventually dissolve as individuals settle into relatively stable sets of relationships. Because network evolution depends on previous network states^20^, the distribution of ties that results from this churn process might help to explain the network’s subsequent evolution. If so, an individual’s churn experience could provide expectations about future outcomes, which is important because social networks influence phenomena as diverse as academic success^21,22^, voting behavior^23^, and cognitive health^24,25^.

## Results

To examine the concept of churn, we fielded a daily survey in a real-world social setting that collected granular data on the network emergence process. A key advantage of this survey was the observation of the network from the point of inception, which is often difficult to observe in nature. In the summer of 2018, 76 individuals undertook a four-week training program in a geographically secluded location in North America. Participants were housed together, and the schedule included both structured daily workshops and unstructured social time. Individuals ranged from graduate students to university faculty members and professional researchers from at least 22 countries. We asked participants to report the other program participants with whom they interacted each day—including the time duration of those interactions – from day 1 through 26, the final day of the program. Further, the program included a mandatory group research project designed to cumulate in a co-authored scientific paper due one year later. Therefore, in addition to the daily interaction network, the data include a behavioral measure of scientific collaboration during and after the summer program. Importantly, only 1.1% of possible dyadic pairs knew each other prior to the program. The data thus describe the emergence and evolution of new, previously unobserved social and scientific collaboration networks in a fixed population of scientists.

**Figure 1:**
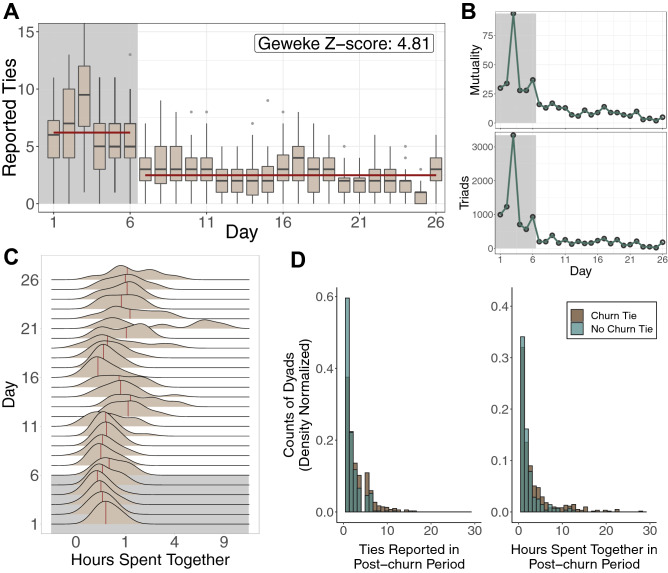
Interperiod differences. (**A**) The churn period (shaded grey) displays higher mean, median, and variance of ties. The figure counts ties from the perspective of alters. For example, if one individual reports one tie to three alters, the ego has three ties but the alters each have one tie. The plot displays the latter number. (**B**) The churn period displays higher counts of mutually reported (i.e. reciprocated) ties and closed triads. We assess whether mutuality and transitivity in the network are different from chance using a conditional uniform graph test^27^. The results show that levels of mutuality and transitivity are present at statistically significant rates ($p < .05$), with the exceptions of mutuality on days 23 and 26. (**C**) Time spent together increases over time. (**D**) Dyads who reported at least one tie during the post-churn period, plotted according to whether that dyad reported a tie during the churn phase. The left panel displays density normalized counts of ties reported during the post-churn period. The right panel displays density normalized hours spent together during the post-churn period.

First, we investigate patterns of interaction over time. If the network emerges gradually according to constant growth processes, then the network emergence process would be consistent with extant models. However, if patterns of interaction in early periods differ substantially from later periods, then extant models are unlikely to explain network emergence. We apply Geweke’s diagnostic to the network’s density over time, which uses a standard Z-score, $z = \frac{{\bar{X}}_1 - {\bar{X}}_2}{\sqrt{ var(X_1) + var(X_2)}}$, to test for the equality of means between two segments of a Markov chain^26^. We find a significant change in density after day 6 and therefore treat days 1–6 and days 7–26 as the churn and post-churn periods, respectively. Figure 1(A) displays higher mean, median, and variance in reported tie counts during the churn period (shaded grey) compared to the post-churn period (t-test: ${\bar{x}}_{1:6} = 6.21$, ${\bar{x}}_{7:26} = 2.47$, $p < .01$, $t = 24.46$; Mood’s test: ${{\tilde{x}}}_{1:6} = 6$, $\tilde{x}_{7:26} = 2$, $p < .01$, $\chi ^2 = 469.63$; F-test: $s^2_{1:6} = 9.78$, $s^2_{7:26}=2.98$, $p < .01$, $F = 3.28$). Further, these differences in interactions appear to be a network-wide phenomenon. Figure 1(B) reports temporal counts of mutually reported ties and closed triads. Churn period ties are highly reciprocal and clustered, which points to an initial process of participants meeting other participants at a rate substantially higher than they sustain in the future. Naturally, these differences suggest a temporal change in the tie generating process.

Temporal differences in tie counts, mutuality, and clustering reveal important variation in tie formation behavior, but these statistics do not necessarily capture tie strength or durability. Figure 1(C) displays the distributions of interaction frequency over time. Mean time spent together increases (t-test: ${\bar{x}}_{1:6} = 24$ mins, ${\bar{x}}_{7:26} = 61$ mins, $p < .01$, $t = 3.65$, comparing the means of daily means), which suggests that individuals settle into more meaningful relationships over time. Further, Fig. 1(D) plots counts of dyads who met during the churn period and in turn reported a post-churn tie compared to dyads who did not meet during the churn period but later reported a post-churn tie. The left panel illustrates that dyads who met during the churn period report higher numbers of subsequent interactions compared to dyads who did not meet during the churn period (t-test: ${\bar{x}}_\mathsf{tie} = 3.20$, ${\bar{x}}_\mathsf{no \ tie} = 2.08$, $p < .01$, $t = 8.91$; KS test: $p <.01$, $D = .20$; Mood’s test: $\tilde{x}_\mathsf{tie} = 2$, ${{\tilde{x}}}_\mathsf{no \ tie} = 1$, $p < .01$, $\chi ^2 = 40.83$). The right panel illustrates that dyads who met during the churn period report more time spent together during the post-churn period (t-test: ${\bar{x}}_\mathsf{tie} = 153$ mins, ${\bar{x}}_\mathsf{no \ tie} = 85$ mins, $p < .01$, $t = 6.10$; KS test: $p < .01$, $D = .14$; Mood’s test: ${{\tilde{x}}}_\mathsf{tie} = 62$ mins, ${{\tilde{x}}}_\mathsf{no \ tie} = 41$ mins, $p < .01$, $\chi ^2 = 11.77$). These results suggest that dyads who met during the churn period in turn display greater durability and strength of interaction during the post-churn period relative to dyads who first met after the churn period. These descriptive results indicate that tie formation behavior and meaningfulness of interaction varies across the churn and post-churn periods.

Next, we examine whether these differences are important for the network’s evolution. If the churn period fails to explain variance in later evolutionary periods, then we can ignore the churn process. To model the network’s evolution, we use a temporal extension of the exponential random graph model ([T]ERGM). ERGMs are a popular family of inferential network models that, in the most basic form, model the probability of a tie in an outcome network as a function of exogenous (e.g. nodal attributes) and endogenous (e.g. homophily) parameters^28–30^. In the cross-sectional case (i.e. a single outcome network at one point in time), the ERGM treats the outcome network as a single multivariate observation and estimates the specified parameters via maximum likelihood. The TERGM is an extension of the ERGM for longitudinally observed networks in discrete time^31^. The TERGM defines the probability of a network at time *t* as a function of covariates at time *t*, as well as dependencies on networks in previous time periods, and then pools estimates over all time periods to compute the probability of the time series of networks^32^. We use maximum pseudolikelihood to estimate the parameters with bootstrapped confidence intervals^33^. The coefficients can be interpreted as the log-odds of forming a tie, akin to logistic regression coefficients.

**Figure 2:**
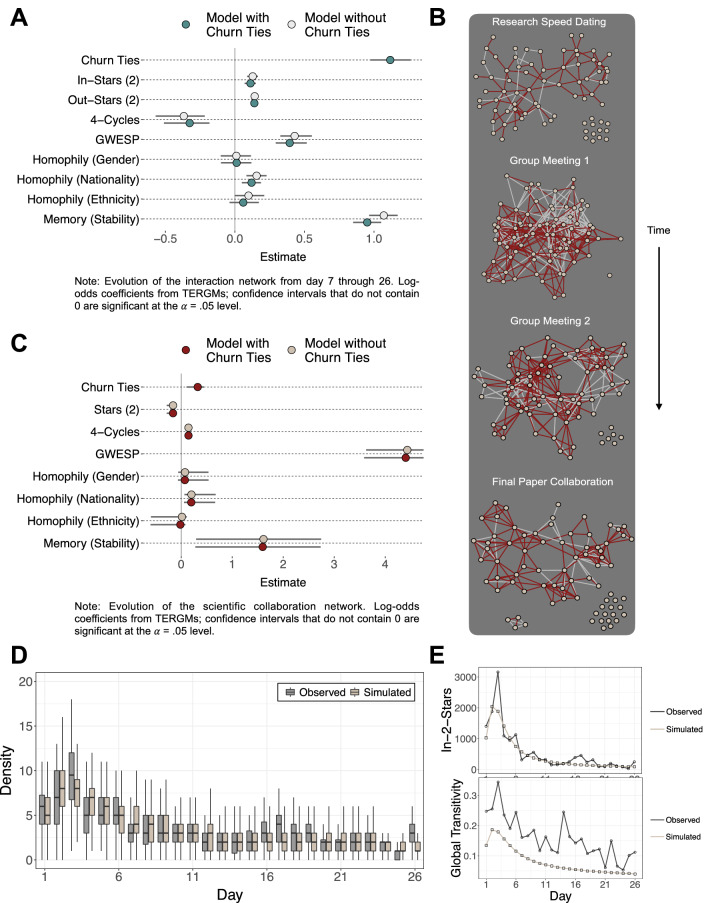
Inference and simulations. (**A**) TERGM results indicate that churn ties help to explain the interaction network’s evolution over days 7 through 26. The plot omits the model’s edges term for ease of visualization. (**B**) The evolution of the scientific collaboration network. Tie color represents the presence (red) or absence (white) of a churn tie in the interaction network. Research speed dating occurred during the second day of the program, followed by group meetings in weeks 2 and 3 and a final paper submitted within one year of the program. The percentage of churn ties are as follows: $N_\mathsf{1} = 75.0\%$, $N_\mathsf{2} = 64.1\%$, $N_\mathsf{3} = 72.9\%$, $N_\mathsf{4} = 75.3\%$). The network diagrams are generated using the statnet package (version 2019.6) in the R statistical programming environment^35,36^. (**C**) TERGM results indicate that churn ties help to explain the scientific collaboration network’s evolution. The plot omits the model’s edges term for ease of visualization. (**D**) 1000 density distributions simulated from the model closely approximate the interaction network’s observed density distributions. (**E**) Counts of 2-stars simulated from the model closely approximate the interaction network’s observed counts of 2-stars, but the model under-predicts at period 3. Global transitivities in the simulated networks follow the same trend as the transitivities observed in the data, but the model consistently under-predicts actual transitivity level. Confidence intervals become too narrow to visualize as the number of simulations increases. Thus, these trend lines represent the statistic counts to which the model converges.

Figure 2(A) presents the results of two TERGMs fitted to the post-churn period (i.e. days 7 through 26). In addition to dyadic interactions on the day (i.e. an “edges” term, omitted for ease of visualization), the models contain structural and homophily-based variables that often influence network evolution. In the models, in-two-stars are motifs in which directed ties exist from nodes *j* and *k* to node *i*, but no tie exists between *j* and *k*, which provides a measure of popularity^30^. Out-two-stars are motifs in which directed ties exist from node *i* to nodes *j* and *k*, but no tie exists between *j* and *k*, which provides a measure of sociality. Four-cycles model the presence of “square” motifs, namely a cycle of directed ties *ij*, *jk*, *kl*, and *li*. The geometrically weighted edgewise shared partners (GWESP) term captures triadic closure (i.e. clustering) in the network. The homophily terms capture the propensity for ties to form between two individuals *ij* who are both male (or non-male), American (or non-American), and white (or non-white), respectively^34^. The model’s “memory” term includes all ties present and absent in the previous day, which accounts for tie autoregression and temporal stability in the network’s ties. Finally, the first model includes ties observed during the churn period as a predictor (our primary parameter of interest), whereas the second model omits this information.

The results indicate that, on average, dyads who met during the churn period are 3 times more likely to report a tie during the post-churn period [$\exp (1.12) = 3.06$]. The coefficients on other model predictors do not appreciably change with the addition of churn ties as an explanatory variable. This stability suggests that churn ties help to explain variance independent of traditional tie formation predictors. This information would otherwise be unavailable had the network been observed only after participants’ initial interactions. Furthermore, the coefficient on the model’s memory term varies little with the addition of churn ties. This suggests that churn ties explain variance beyond the simple presence of tie autoregression.

Although these results suggest that churn ties help to explain the network’s evolution, a natural question is whether these churn ties explain disproportionate variance in subsequent tie formation, relative to ties observed in any given period of the program. In the SI, Tables S3-S4 report evidence that churn ties explain disproportionate variance in subsequent tie evolution. When tie weights contain raw counts of interactions, each churn tie in a given dyad displays a larger log-odds coefficient than ties observed during any given period that precedes a wave of the interaction network, that is, the observed network at time *t* (0.54, 95% CI [0.48, 0.58] vs. 0.33, 95% CI [0.30, 0.37]; $z = 6.67$). When tie weights instead contain counts of interactions normalized by the number of waves of interaction, the coefficient on all ties becomes larger than the coefficient on churn ties (3.22, 95% CI [2.90, 3.50] vs. 5.49, 95% CI [4.91, 6.25]; $z = 6.03$). However, this result implies that each churn tie explains $3.22 / 5.49 = 59\%$ of subsequent interaction tie formation. Thus, regardless of transformation, ties observed in the first six days of the program alone in turn explain the majority of subsequent tie formation.

These results indicate that the churn process helps to explain the interaction network’s evolution. However, social networks also powerfully influence individuals’ outcomes beyond daily interactions. We next examine whether the churn process is related to subsequent behavioral outcomes among participants, particularly their collaboration on scientific papers. The summer program included self-organized opportunities for participants to exchange research interests and form project groups. The program’s second day included a “research speed dating” session for participants to discuss research interests. The program’s second and third weeks included two group meetings to form final project groups. In turn, groups submitted an optional, co-authored scientific paper within one year of the program. Because 39 individuals worked on multiple final papers, these collaborations constitute a co-authorship network.

Figure 2(B) plots the evolution of the collaboration network. Collaboration tie color represents the presence (red) or absence (white) of a churn tie in the interaction network (i.e. whether those individuals met during the first 6 days of the program). Although the majority of collaborators met at least once during the churn period ($N_\mathsf{1} = 75.0\%$, $N_\mathsf{2} = 64.1\%$, $N_\mathsf{3} = 72.9\%$, $N_\mathsf{4} = 75.3\%$), research ties between individuals who did not meet during the churn period dissolved with greater frequency. From group meeting one to the final paper submission, 36.4% of churn ties dissolved and 62.6% of non-churn ties dissolved (z-test: $\chi ^2 = 20.84$, $p < .001$). Therefore, individuals who met during the churn period formed more durable co-authorship ties compared to individuals who did not meet during the churn period.

To add inferential precision, Fig. 2(C) presents the results of two TERGMs fitted to the scientific collaboration network’s evolution. The first model includes churn ties as a predictor, and the second model omits these ties as an explanatory variable. The other covariates in the model mirror those used in the interaction network model presented above, with the exception that we use the undirected versions of these statistics, because the co-authorship network contains undirected ties. Undirected two-stars are motifs in which an undirected tie exists between node *i* and node *j*, as well as node *i* and node *k*, with no tie between *j* and *k*. A negative coefficient on this statistic would indicate that unclosed triads are relatively unlikely, which would be consistent with previous work that finds high levels of clustering in co-authorship networks^37^. Four-cycles (i.e. square-like motifs) capture the propensity for two individuals *i* and *k* to have the same co-authors *j* and *l*, but to not be co-authors themselves. GWESP models the tendency towards triadic closure in co-authorship patterns. The homophily parameters model the possibility that individuals who share certain characteristics are more likely to co-author. The memory term captures temporal stability in research ties, namely that collaboration ties at time *t* depend on whether or not the individuals were research collaborators at time $t-1$.

The results indicate that, on average, dyads who met during the churn period are 1.38 times more likely to form scientific collaboration ties [$\exp (0.32) = 1.38$]. This result is noteworthy, because the model’s memory term contains all collaboration ties present and absent in the previous period of the collaboration network. Thus, churn ties explain variance in project collaboration independent of the research groups themselves. Coefficients on other predictors in the model do not appreciably change with the addition of the churn ties. Thus, churn ties provide explanatory information independent of other known co-authorship predictors, such as clustering^38,39^. Together, these results imply that an individual’s churn experience provides explanatory leverage on scientific collaboration as late as one year after the program.

Although these results suggest that churn ties help to explain the scientific network’s evolution, we also examine whether churn ties explain disproportionate variance in subsequent tie formation. In the SI, Tables S5-S6 report evidence that churn ties explain disproportionate variance in subsequent tie evolution. When tie weights contain raw counts of interactions, each churn tie in a given dyad displays a larger log-odds coefficient than ties observed during any given period that precedes a wave of the scientific network (0.20, 95% CI [0.18, 0.23] vs. 0.11, 95% CI [0.06, 0.21]; $z = 2.27$). When tie weights instead contain counts of interactions normalized by the number of waves of interaction, the coefficient on all ties becomes larger than the coefficient on churn ties (1.20, 95% CI [1.08, 1.36] vs. 2.22, 95% CI [1.48, 3.11]; $z = 2.61$). However, this result implies that each churn tie explains $1.20/2.22 = 54\%$ of subsequent scientific tie formation. Thus, regardless of transformation, interaction ties observed in the first six days of the program alone in turn explain the majority of subsequent scientific tie formation.

Finally, we use the observed interaction network data to introduce a model of the churn process. For a fixed level of sociality, dynamics during the churn versus post-churn periods should differ for at least two reasons. First, individuals exhibit some fixed social bandwidth above which the maintenance of relationships is physically or psychologically untenable^40–42^. Thus, individuals should form relationships up to some threshold and dissolve ties above that threshold. Second, under conditions of the emergence of a new network, some time cost accompanies the possibility of tie formation. During the earliest iterations of tie formation, the fewest individuals know each other. Therefore, the greatest number of potential connections exist. As individuals settle into friendships and cliques, however, a node’s set of available social worlds begins to shrink. Here, we operationalize these social bandwidth and time cost parameters for a fixed level of sociality.

i. Choose some fixed number of nodes, *V*.
ii. Choose some fixed level of sociality, i.e. a fixed probability of tie gain, $p_\mathsf{gain}$.
iii. Choose some time cost, $\alpha$, such that nodes lose ties with $p_\mathsf{loss_t} \sim \mathsf{bin}(1, \ p_t)$ where $p_t = (T - t)^{-\alpha }$, $0< \alpha < 1$, $t \in \{ 1,\dots ,T \}$, and *T* equals the total number of time slices in the network sequence.
iv. Choose some fixed social bandwidth, *b*, $0 \le b \le 1$, that limits the number of possible ties, $b(V - 1)$, that a given node can maintain. Nodes with degree (number of ties) below this limit form new ties with probability $p_\mathsf{gain}$ and lose existing ties with probability $p_\mathsf{loss_t}$. Above this limit, nodes lose ties with probability $p_\mathsf{loss_t}$ with no opportunity for tie gain.

Figure 2(D) plots density distributions from 1000 model simulations for parameters $V=76$, $\alpha = 0.45$, $p_\mathsf{gain} = 0.07$, and $b = 0.21$. We optimize on observed network density to estimate these parameters. The simulations closely approximate the observed density distributions. Figure 2(E) displays the simulation results for two-stars and triads. The model closely approximates the observed two-stars but under-predicts global transitivities. Figure S9 provides additional model simulations, which illustrate that higher levels of sociality, higher social bandwidths, and lower time costs produce networks with higher densities, all else equal.

Together, our empirical results suggest that tie formation behavior during the earliest periods of network emergence may differ significantly from later evolutionary periods. These results are consistent with a previous study of a large-scale online game network, which displayed large differences in tie formation and dissolution probabilities in earlier versus later evolutionary periods [Fig. S3 in Ref. ^1^]. Further, the churn process helps to explain the interaction network’s evolution, as well as the evolution of scientific collaboration during and after the program. These results are consistent with recent work that finds that integration into an emerging student network explains academic performance^22^ (see also Ref. ^21^). However, our results also suggest that the very earliest interactions between individuals are key to understanding the evolution of the emerging social network and behavioral outcomes at later time periods. Finally, whereas previous work finds evidence of ego-level tie distribution stability after entering a new social setting^42^, our study provides a finer-grained-in-time analysis of the emergence and evolution of those new ties, finding in particular that the earliest ties disproportionately explain variance in later interaction patterns, as well as collaboration in the scientific network. Whereas co-authorship networks represent an important area of previous network scientific inquiry^37–39^, our study provides empirical results and a theoretical model that demonstrate the ways in which those co-authorship networks can emerge.

The proposed concept and model of churn narrows the scope of the network emergence problem, particularly in new networks marked by an initial cycle of rapid tie expansion and contraction. This concept may apply to a broad cross-section of new social situations, but a more developed taxonomy of the dimensions that constitute “new” networks would prove fruitful. We emphasize that this study’s empirical component relies on one case of network emergence, in which interaction ties and scientific collaboration ties evolve over time. We exercise caution about drawing generalizations from a single case of network emergence. For example, planned events at the summer program may exert structural influence on interactions beyond the simple presence of self-organized interactions. The program may also be unique, because the setting included both social and professional, research-based components. The computational results from our theoretical model suggest that this process could unfold in contexts in which individuals face time costs (i.e. the set of potential alters shrinks over time) and display social bandwidths (i.e. a limit exists on the number of relationships an ego can feasibly maintain). However, follow up studies that examine the emergence of other networks will help to clarify the extent to which the concept of churn generalizes across contexts and whether structured events or activities moderate the findings.

In addition, future work might consider processes of community formation and evolution in emerging networks, such as the extent to which the earliest community ties exert disproportionate influence on subsequent meso-structural evolution and whether those ties influence or depend on relationships in other emerging or established network layers^43,44^. Second, cooperation evolution research might gain from attention to the ways in which the earliest periods of network formation affect the chances for future cooperation^45^. For example, previous work finds that excessive ties can hinder cooperation^46^, and our results suggest that the churn period might provide insights into this tie rewiring and dissolution process as the network moves towards more stable evolution. Third, the question of network emergence might benefit from investigation via Bayesian frameworks of network evolution, both theoretically—such as the extent to which nodes disproportionately learn from and update ties according to the nodes’ earliest experiences^47^—as well as methodologically, such as inferential models that use the earliest network interactions to predict later interactions^48,49^. Finally, future work would benefit from random assignment procedures to better isolate underlying tie formation mechanisms, attention to contexts in which node entrance and exit are possible, and extension of the model presented here. For example, the model accommodates ego- and network-level representations, as well as directed or undirected ties, but could also extend to weighted or signed ties.

### Ethics approval and consent to participate

All protocols in the study adhere to relevant institutional guidelines and regulations. This study was determined exempt by The Ohio State University’s Institutional Review Board (#2018E0343 and #2019E0900). All participants were over the age of 18, and informed consent was obtained from all survey participants.

## Supplementary information

Supplementary material 1
