# Supplementary material for: Dynamics of social network emergence explain network evolution
Source: Sci Rep. 2020 Dec 14;10:21876. doi: 10.1038/s41598-020-78224-2 (PMC7736284; doi:10.1038/s41598-020-78224-2)
Supplement: Supplementary file 1 — Supplementary material 1 [file 41598_2020_78224_MOESM1_ESM.pdf]

Supplementary Information for: Dynamics of Social Network Emergence Explain Network Evolution

Caleb Pomeroy[1], Robert M. Bond[2], Peter J. Mucha[3], and Skyler J. Cranmer[1,*]

[1]Department of Political Science, The Ohio State University, Columbus OH 43210, USA.

[2]School of Communication, The Ohio State University, Columbus OH 43210, USA.

[3]Carolina Center for Interdisciplinary Applied Mathematics, Department of Mathematics, University of North Carolina, Chapel Hill, NC 27599, USA.

[*]To whom correspondence should be addressed. E-mail: cranmer.12@osu.edu

Survey Design and Procedure

As described in the main text, we fielded a daily survey in a four-week training program in North America during the summer of 2018. The survey was hosted on the Qualtrics platform and was accessible online via a daily link disseminated to participants' email addresses, beginning on the first day of the program through the final day, for a total of 26 unique days. Participants could complete up to one survey per day and had the option to opt-out of receiving the study invitation.

The survey instrument begins with a consent form that describes the nature of the study as well as the rewards scheme. Subjects received $3 in Amazon gift card credit for the first survey completed, $1 in credit for each additional survey, and one (1) entry into a raffle for an iPad for each dollar earned. After the consent page, participants selected their name from a dropdown list for the purposes of tracking rewards, and indicated whether they had previously completed the survey. Then, subjects were presented with the prompt "With whom did you interact today? Select as many as you wish," as well as an alphabetical list of names and photos of the students enrolled in the program with tick marks to respond to the prompt. Then, subjects were presented with the prompt "Approximately how many

minutes did you spend interacting with each of the following individuals?” with the alters reported on the previous page presented alongside sliders that ranged from 0 to 600 minutes.

If respondents had previously participated in the survey, then the current survey session terminated. If instead this was the first time that a respondent participated in the survey, the next page presented the prompt “Who at the [summer program] did you know personally prior to the program?” with the alphabetical list of students presented again. Finally, demographic questions were collected at the end of survey, including participants’ stage in education and university department, whether the participants were assigned a roommate during the summer program, research interests, hobbies, gender identification, age, ethnicity, and nationality. For first time respondents, the survey terminated after this demographics page. The average earnings for participation in the study were $8.90, with 8.9 entries into the iPad raffle. Median time spent on each survey was approximately 4 minutes and 48 seconds.

Here, we present an anonymized version of the summer program’s schedule to provide details about the nature of the program. Weekdays of the program consisted of training workshops. The program was four weeks in duration. The optional research speed dating session took place during the first week of the program. The first group project meetings took place from the middle of the second week to the early part of the third week of the program. The second project meetings were scheduled from the end of the third week of the program to the early part of the fourth and final week of the program. Each team was required to have one "first" meeting and one "second" meeting. The program concluded with group research presentations. Group research papers were an optional task that groups could submit within the following year in order to receive an official certificate of participation in the program.

The scientific collaboration networks were constructed from publicly available information available through the summer program’s website. During the program, participants signed up for intellectual speeding dating and group meeting slots via the program’s website. In turn, co-authored papers were completed and published on the program’s website one year after the program. We note that this scien-

tific collaboration network contains isolates (i.e. unconnected nodes). Since the research speed dating session was an optional event, the isolates represent individuals who did not participate in the event. Similarly, since the final co-authored paper was an optional task, isolates reflect those individuals who did not complete a final paper at the expense of a program certificate. Finally, although the two remaining waves of the scientific network (i.e. "Group Meeting 1" and "Group Meeting 2") were mandatory sessions, one participant in the first meeting and seven participants in the second meeting experienced difficulties accessing the system in order to formally sign up for the event. Because we rely on this online system to construct these networks, we do not have access to information about the groups in which these individuals participated, or whether they participated in the event at all. However, because our scientific collaboration models estimate the effect of interaction ties on scientific collaboration ties, it is likely the case that, if anything, these missing observations artificially suppress rather than inflate our estimates.

Relationship Between Planned Activities and Dyadic Interactions

Here, we consider whether findings from the interaction network, such as the churn process or an increase in mean time spent over time, reduce to interactions in the scientific collaboration network.

We note that the high levels of reported interactions during the first week of the program are unlikely to be a function of unusually high numbers of planned activities during the first week of the program. In particular, if the research speed dating session caused individuals to report many contacts during the first week of the program, then our churn effect is simply a function of interactions in the scientific collaboration network. To check for this possibility, we calculated the number of research speed dating ties that overlapped with ties in the interaction network. Of the 1,550 unique interaction ties reported during days 2-6, 43 of those ties, or 2.77%, were present in the speed dating session. Fig. 2(B) in the main text shows that the speed dating event was also somewhat under-attended relative to subsequent

research events, which increases our confidence that the high levels of participant interactions found during the first week is not simply a result of unusual activities specific to the first week of the program.

Further, we consider whether planned research group meetings are the cause of dyads spending longer time together in the post-churn period. During the time range of group meeting one (time slices 9-15 in our data), participants reported a total of 53,322 minutes spent with alters (and 648 ties), of which 38,967 minutes (and 507 ties) were spent with individuals who were not an ego research collaborator, and 14,355 minutes (and 141 ties) were spent with individuals who were an ego research collaborator. Thus, 38,967/53,322 = 73.1% of total time was spent with non-collaborators, and 14,355/53,322 = 26.9% of time was spent with individuals with whom the individual was collaborating on a research project. These numbers suggest that the majority of an ego's time is spent with individuals who were not research collaborators. However, 14,355/141 = 102 minutes were reported per collaborator tie and 38,967/507 = 77 minutes were reported per non-collaborator tie. Thus, the average participant spent a total of ∼25 minutes more time with collaborators during this time period, which essentially captures the meeting that occurred during that time period. Indeed, it would be surprising if group members spent no more time with each other during this meeting period.

These results are similar during the time period of the second group meeting (time slices 19-22 in our data). Participants reported a total of 32,383 minutes spent with alters (and 445 ties), of which 24,355 minutes (and 356 ties) were spent with non-collaborators, and 8,028 minutes (and 89 ties) were spent with individuals who were a research collaborator. Thus, 24,355/32,383 = 75.2% of total time was spent with non-collaborators, and 8,028/32,383 = 24.8% of time was spent with research collaborators. Again, the majority of an ego's time is spent with alters with whom the ego was not collaborating on research. However, egos reported about 8,028/89 = 90 minutes spent per tie with collaborators, and 24,355/356 = 68 minutes spent per tie with non-collaborators. Thus, the average participant reported a total of about 22 minutes more time spent with research collaborators, which

essentially captures the mandatory group meeting that occurred during this time period.

In sum, subjects reported slightly more time spent per tie with fellow group members during these time periods. However, time spent with collaborators accounts for a relatively small proportion (usually around a quarter) of total time spent with other individuals at the summer program. Therefore, our finding that dyads spend more time together in the post-churn period does not appear to reduce to the planned research meetings.

A related concern is whether the interaction ties reported in Fig. 1(D) primarily correspond to ties within research groups. Here, we compare the counts of ties reported to research collaborators versus counts of ties reported to non-collaborators during the post-churn period (i.e. time slices 7-26). Because the research teams evolved, we conduct separate comparisons for group meeting one members and group meeting two members. Using group meeting one as the collaborator versus non-collaborator node set, we find that participants reported an average of 13.7 ties to group members and 35.7 ties to non-group members during time periods 7-26. Thus, participants reported significantly more ties to non-group members (t-test: $p < .01$, t = -2.95). Similarly, using group meeting two as the collaborator versus non-collaborator node set, we find that participants reported an average of 12.1 ties to group members and 37.3 ties to non-group members. Thus, participants reported significantly more ties to non-collaborators (t-test: $p < .01$, t = -3.36).

Furthermore, we consider whether the extreme events (i.e. those dyads who very frequently interacted during the program) primarily correspond to research collaborators. Here, we apply a Kolmogorov-Smirnov test to the distributions of tie counts according to collaborators versus non-collaborators. We find no significant differences at the $\alpha = .05$ level, using various cutoffs for "extreme events." For example, we find no significant differences when using group meeting one as the collaborator versus non-collaborator node set and subsetting the tie set to more than five dyadic interactions (p-value = 0.07, $D = 0.21$) or more than 10 dyadic interactions (p-value = 0.46, $D = 0.35$). Similarly, we find

no significant differences between the distributions when using group meeting two as the collaborator versus non-collaborator node set and subsetting the tie set to dyads who reported more than five interactions (p-value $= 0.17$, $D = 0.18$) or more than 10 interactions (p-value $= 0.35$, $D = 0.38$). In short, the extreme events (i.e. dyads who frequently interact) do not appear to reduce to research collaboration.

Participant Descriptive Statistics

Figure S1 displays self-reported demographic characteristics for the sample. The average participant was a white, male PhD student in his 20s from North America or Europe. Although the sample is not representative of the broader US population, the sample provides three desirable features for the study of network emergence. First, the program was geographically secluded, which helps to minimize potential interference from socialization patterns outside of the program, i.e. the population was fixed. Second, only 1.1% of possible dyads knew each other prior to the program, which ensures that we are observing the emergence of a new social network. Finally, detailed below, participation in the study did not systematically vary by demographic characteristics.

Participation Rates

The summer program included 78 students. Two students elected to opt-out of the study, which resulted in a total sample size of 76 subjects. Of those 76 students, 61 students took the survey at least once. The maximum number of participants took place on day 3, with 30 participants. The minimum number of participants took place on day 13, with 7 participants. Days 3 and 5 saw the maximum number of alters nominated at least once, with all 76 participants receiving at least one nomination. Day 25 saw the minimum number of alters nominated at least once, with 48 alters nominated. Although the churn period (days 1 through 6) saw the highest average number of alters nominated ($\bar{x} = 75$), more

importantly, we retain adequate survey coverage of the network over time. For example, an average of 66 alters were nominated during periods 7 through 26. During the final 10 periods, 65 alters were nominated at least once on average. This implies that survey coverage of the network does not change simply due to attrition over time. Further, Fig. S2(C) considers the percentage of individuals who either participated or were nominated by an alter. Nearly every individual appears in our data as either an ego or an alter in each time slice, which increases confidence that the survey was not systematically missing a portion of the network. Further, 85.7% of individuals present in at least one churn period dyad (either as an ego or alter) in turn participated at least once during the post-churn period. Because we emphasize differences between these two periods, this proportion suggests that the survey maintains a relatively high level of coverage beyond the churn period.

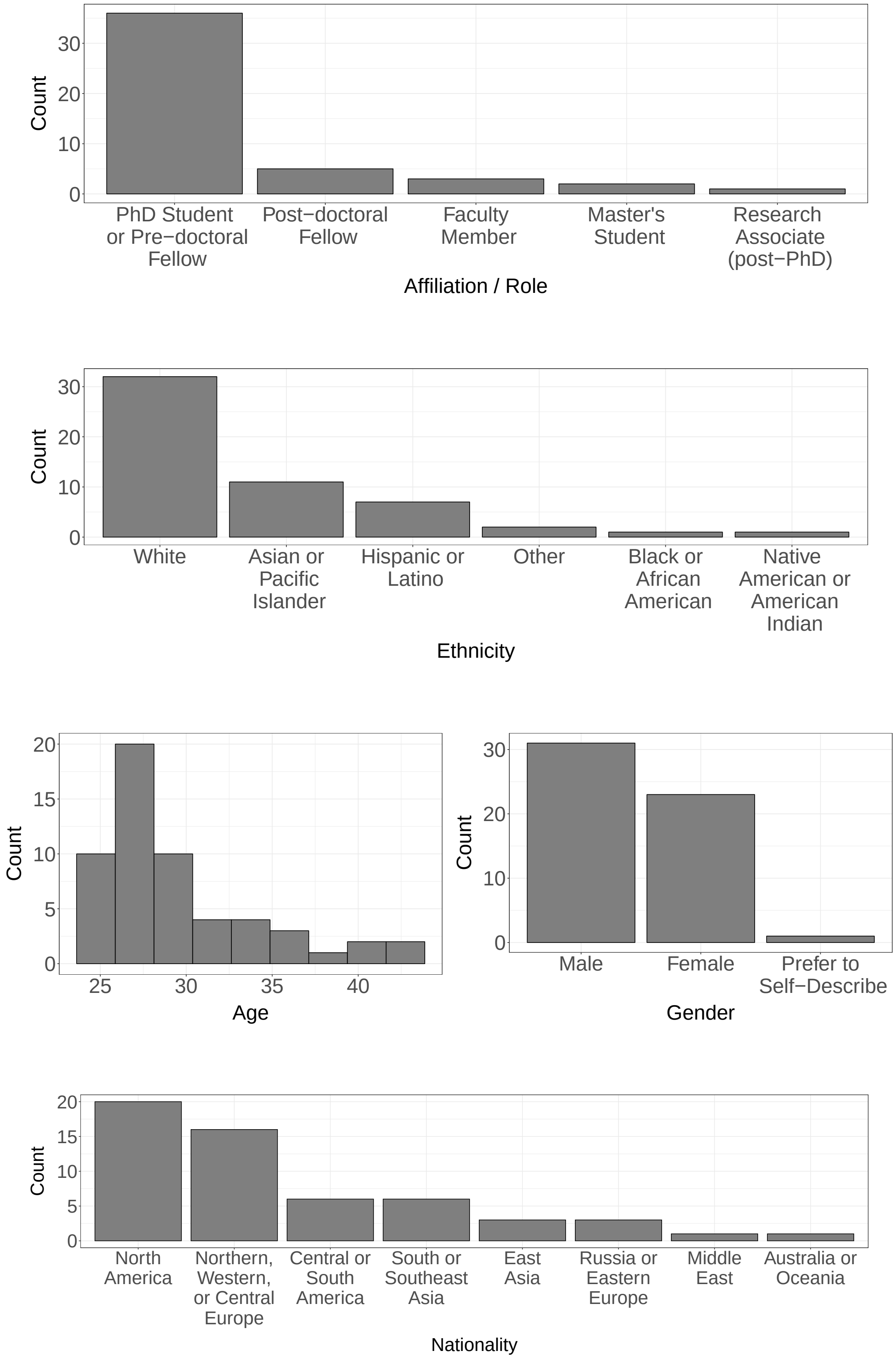

Figure S1: **Participant Descriptive Statistics.** Top row displays counts of participants by role within their respective university or research organization. Second row displays counts of participants by ethnicity. Third row (left) displays counts of participants by age. Third row (right) displays counts of participants by gender. Bottom row displays counts of participants by nationality, grouped into regions. Plots here and in the main text were generated using the 'ggplot2' package (1) in the R statistical computing environment (2).

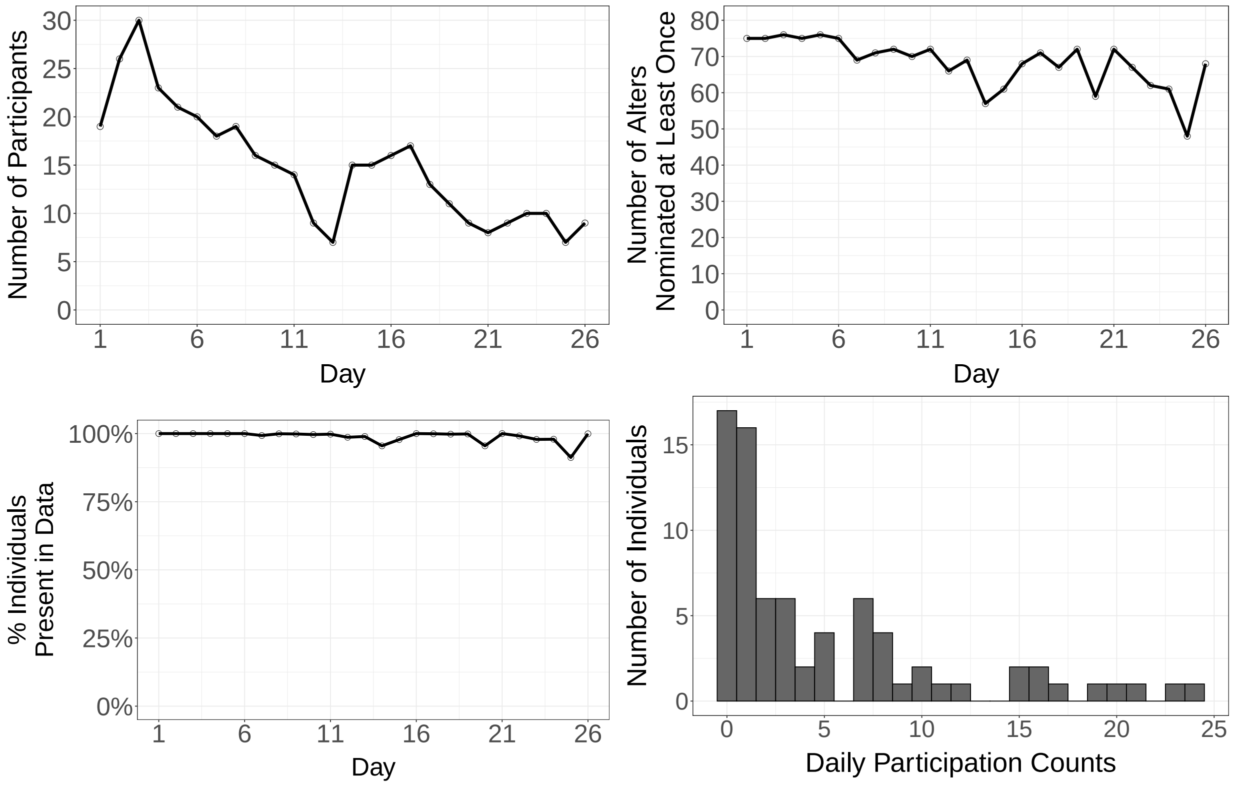

Figure S2: **Participation Counts and Alters Nominated.** Top left plot displays the number of survey participants on each day. Top right plot displays the number of alters nominated at least once by an ego participant on each day. Bottom left plot displays the percentage of individuals in the program present as either an alter or an ego in the survey data on each day. Bottom right plot displays the number of individuals (the y-axis) who participated X times (the x-axis).

Participation by Demographic Characteristics

Table S1 displays linear models that regress participation rates on demographic characteristics. These models are subsetted according to the churn period (days 1-6) and the post-churn period (days 7-26). Because our paper emphasizes differences during these two periods, it is important to ensure that differences in these periods do not result simply due to systematic changes in participation. In Models 1 and 3, the outcome variable represents whether or not an individual participated at least once during the respective time period, estimated by logistic regression. In Models 2 and 4, the outcome variable represents counts of surveys submitted (i.e. the number of times an individual participated during the respective periods) estimated by poisson regression. The models contain 72 observations, because 4 individuals opted to withhold demographic information. We find that participation rates do not systematically vary by demographic characteristics, both in terms of the decision to participate at least once and in counts of surveys submitted. One exception is that non-American respondents completed approximately 1.32 times the number of surveys as American respondents during the post-churn period.

Table S1: Participation by demographic characteristics.

	(1) Participated (Churn)	(2) Participation Counts (Churn)	(3) Participated (Post-churn)	(4) Participation Counts (Post-churn)
Intercept	3.19	1.46	−1.19	0.85
	(2.13)	(0.76)	(2.06)	(0.52)
Age	−0.10	−0.03	−0.00	0.01
	(0.06)	(0.02)	(0.06)	(0.01)
Graduate Student	−0.31	0.08	0.86	0.08
	(0.70)	(0.25)	(0.74)	(0.18)
Non-male	0.16	0.10	0.26	−0.21
	(0.51)	(0.18)	(0.51)	(0.13)
Non-white	−0.70	−0.37	−0.75	−0.22
	(0.53)	(0.19)	(0.54)	(0.14)
Non-American	0.51	0.25	0.86	0.28^{*}
	(0.52)	(0.19)	(0.53)	(0.14)
Num. obs.	72	72	72	72

$^{***}p < 0.001$, $^{**}p < 0.01$, $^{*}p < 0.05$

Note: Models 1 and 3 display logistic regression results with an outcome variable that indicates whether or not an individual submitted at least one survey during the relevant time period. Models 2 and 4 display poisson regression results with an outcome variable that represents counts of surveys submitted during the respective time period. The lack of significance here indicates that participation does not systematically vary by demographic characteristics.

Full Inferential Results and Robustness Checks

In the main paper, we present descriptive differences of the interaction network across time, as well as inferential results that indicate that churn ties explain evolution of the interaction and scientific collaboration networks. Here, we present further descriptive results and full inferential results for the interaction network, including model robustness checks.

Figure S3 presents all possible combinations of 3 nodes in the network and contrasts the number of ties between these nodes during the churn and post-churn periods. The lower triangle displays higher counts than the upper triangle, which indicates dissolution of the earliest ties as the network evolves. For example, closed triangles during the churn period dissolve to 3 isolates in the post-churn period 4 times more often than the reverse: 3 isolates during the churn period forming a closed triangle during the post-churn period. Further, 2,956 closed triangles from the churn period remain closed triangles during the post-churn period, which can be thought of as a durable social backbone (3) to the network's post-churn evolution. These results suggest that the network "settles" after an initial, rapid expansion of ties.

Figure S4 further illustrates this process of rapid tie formation followed by tie dissolution at the ego-level. Here, we calculate the distributions of degree and betweenness centralities at the ego-level over time, subsetted into four weekly periods. While Fig. 1(A) in the main paper displays the network's density over time, these measures of centrality allow us to further demonstrate that high levels of initial interaction were not unique to a handful of high-interaction nodes or clusters of egos: the churn process extends throughout the network. The earliest periods of network emergence display relatively flat distributions of centralities (relative to later periods), which is consistent with our broader findings that the emerging network goes through an initial period of high levels of interaction. As the program progresses, however, relatively skewed centrality distributions emerge, consistent with distributions that

Ties for All 3 Node Combinations

Stability Period

Churn Period		0	1	2	3	Total
	0	6816	3282	1894	314	12306
	1	7651	7841	6117	1579	23188
	2	4911	7220	8487	3314	23932
	3	1315	2454	4149	2956	10874
	Total	20693	20797	20647	8163	70300

Figure S3: **Ties that exist in 3 node combinations.** All possible combinations of 3 nodes, tallied by the number of ties the 3 nodes shared during the churn period (days 1-6) versus the post-churn period (days 7-26). The lower triangle displays higher counts than the upper triangle, which indicates dissolution of the earliest ties over time. The frequencies are significantly different according to a chi-square test $\chi^2(9) = 10214$, $p < .01$.

typically mark more-established social networks, namely that a small proportion of nodes often enjoys an abnormally large number of ties. We can conclude from these results that, in general, the social role of specific egos is less important to the structure of the network in the earliest periods relative to later periods: many egos interact with many alters. However, as the level of network churn declines and the network begins to take more stable form, specific egos play an outsized role in the structure of the network. Thus, the social role of agents becomes more important as the network transitions to more stable periods of evolution.

Table S2 presents the results of TERGMs with varying definitions of the churn period, namely defined as days 1-3, days 1-6 (as in the main text), and days 1-9. As expected, the size of the coefficient on the churn ties increases as more days are included, akin to autoregression. It is noteworthy, however, that even the first 3 days of tie formation proffer explanatory information about the network's subsequent evolution. The coefficients on other model terms are also very stable across these different definitions. These results increase our confidence that the results are robust to changes in churn period definition. All TERGM analyses were conducted using the 'btergm' package (4) in the R statistical

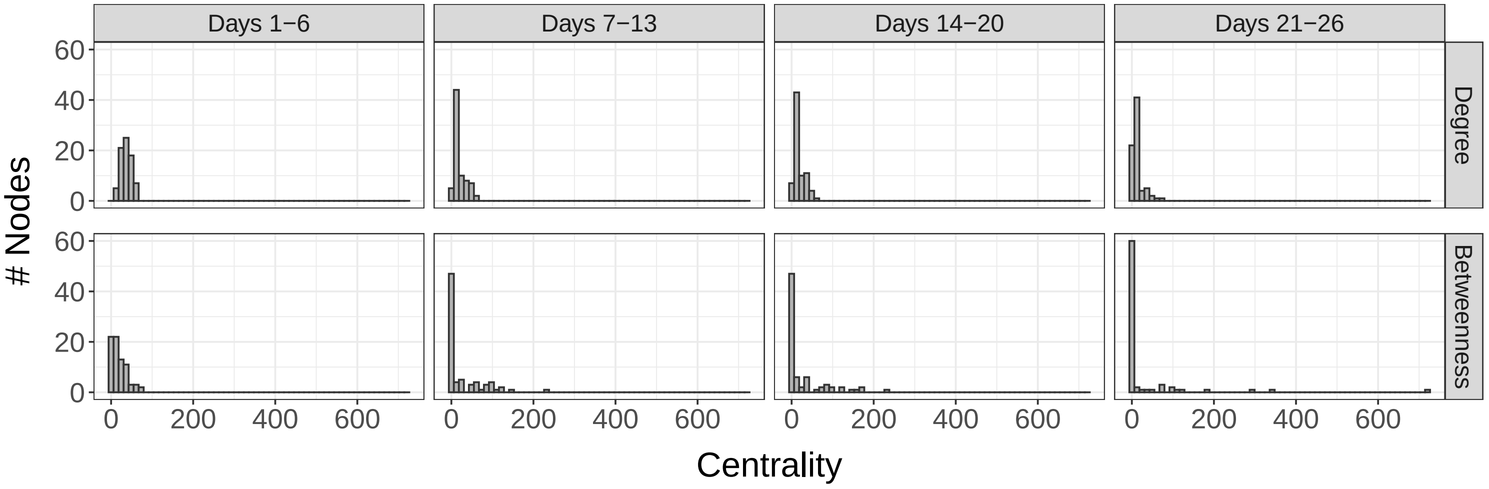

Figure S4: **Ego centralities over time.** Distributions of degree centralities (top) and betweenness centralities (bottom) demonstrate the transition from the earliest periods marked by high levels of interaction across the network to later periods of evolution in which relatively skewed distributions emerge. This process illustrates the emergence of centrality patterns that often mark more-established social networks.

computing environment (2).

Although the above results suggest that the earliest periods of network formation provide explanatory information about the interaction network's subsequent evolution, here we further examine the extent to which those earliest ties explain disproportionate variance in subsequent evolution, above and beyond ties observed in any given period of evolution. To accomplish this task, we compare a model specified with only churn ties to a model specified with all prior interactions that precede a given period t, weighting ties by frequency (i.e. counts) of daily interactions. Table S3 displays the results. The results suggest that ties formed during the churn period alone have a log-odds coefficient of 0.54. Because the ties here are weighted, this coefficient implies that each interaction during the churn period within a given dyad increases the odds of forming a subsequent interaction tie by $\exp(.54) = 1.72$. For example, a dyad with four interactions during the churn period would enjoy ceteris paribus a $1.72 \times 4 = 6.88$ greater odds of a subsequent interaction tie relative to a dyad with no interactions during the churn period. In contrast, all ties observed in the periods that precede a given wave of the interaction network

Table S2: Comparison of different definitions of churn period

	Churn Period Defined as:		
	**Days 1-3**	**Days 1-6**	**Days 1-9**
Edges	-4.66^{*}	-5.01^{*}	-5.37^{*}
	$[-4.78; -4.54]$	$[-5.19; -4.87]$	$[-5.61; -5.18]$
Churn Ties	0.64^{*}	1.12^{*}	1.47^{*}
	$[0.54; 0.73]$	$[0.97; 1.25]$	$[1.28; 1.66]$
In-stars (2)	0.09^{*}	0.11^{*}	0.11^{*}
	$[0.06; 0.13]$	$[0.07; 0.15]$	$[0.06; 0.16]$
Out-stars (2)	0.14^{*}	0.14^{*}	0.14^{*}
	$[0.13; 0.15]$	$[0.13; 0.15]$	$[0.13; 0.15]$
4-cycles	-0.12^{*}	-0.33^{*}	-0.24^{*}
	$[-0.44; -0.06]$	$[-0.52; -0.19]$	$[-0.50; -0.10]$
GWESP (fixed = 0)	0.40^{*}	0.39^{*}	0.36^{*}
	$[0.32; 0.52]$	$[0.29; 0.51]$	$[0.25; 0.51]$
Homophily (Gender)	0.04	-0.02	-0.02
	$[-0.06; 0.13]$	$[-0.13; 0.09]$	$[-0.15; 0.11]$
Homophily (Nationality)	0.15^{*}	0.10^{*}	0.11^{*}
	$[0.07; 0.23]$	$[0.03; 0.17]$	$[0.02; 0.19]$
Homophily (Ethnicity)	0.07	0.07	0.10
	$[-0.01; 0.16]$	$[-0.03; 0.16]$	$[-0.01; 0.21]$
Memory (Stability)	0.95^{*}	0.95^{*}	0.92^{*}
	$[0.85; 1.07]$	$[0.84; 1.05]$	$[0.80; 1.03]$
Num. obs.	30127	23717	18139

Note: Log-odds coefficients from TERGMs on the evolution of the network from days 4-26, days 7-26, and days 10-26, respectively. A star indicates significance at the $\alpha = .05$ level.

(that is, the observed network at time t) have a coefficient of 0.33. We note that the churn tie coefficient is significantly larger than the coefficient on all ties ($z = 6.67$) according to a standard Z-test for regression coefficient equality, $Z = b_1 - b_2 \Big/ \sqrt{se_{b_1}^2 + se_{b_2}^2}$, where b's are the coefficients and se_b's are the coefficients' associated standard errors (5). Together, these results suggest that each churn tie explains approximately $.54/.33 = 1.64$ times more variance in interaction tie evolution than a tie in any given period that precedes a wave of the interaction network.

Table S3: TERGMs: Evolution of the Interaction Network (churn vs. all ties)

	**Model 1**	**Model 2**
Edges	-4.99^{*}	-5.48^{*}
	[−5.15; −4.86]	[−5.71; −5.30]
Churn Ties	0.54^{*}	
	[0.48; 0.58]	
All Previous Ties		0.33^{*}
		[0.30; 0.37]
In-stars (2)	0.11^{*}	0.15^{*}
	[0.07; 0.15]	[0.11; 0.19]
Out-stars (2)	0.14^{*}	0.14^{*}
	[0.13; 0.15]	[0.13; 0.15]
4-cycles	-0.34^{*}	-0.30^{*}
	[−0.52; −0.20]	[−0.48; −0.18]
GWESP (fixed = 0)	0.38^{*}	0.37^{*}
	[0.28; 0.50]	[0.27; 0.49]
Homophily (Gender)	−0.00	−0.00
	[−0.11; 0.10]	[−0.12; 0.11]
Homophily (Nationality)	0.06	0.10^{*}
	[−0.01; 0.13]	[0.02; 0.17]
Homophily (Ethnicity)	0.04	0.04
	[−0.07; 0.15]	[−0.06; 0.16]
Memory (Stability)	0.86^{*}	0.57^{*}
	[0.76; 0.97]	[0.44; 0.67]

Note: A star indicates significance at the $\alpha = .05$ level. Ties are weighted by frequency (i.e. counts) of daily interactions.

The above results provide initial leverage on the extent to which churn ties explain disproportionate variance in subsequent interaction tie formation, relative to ties observed in any given period of the network's evolution. At the same time, the results could be skewed by the fact that the number of waves in the churn period is smaller than the number of waves in all periods prior to t. This larger number of opportunities to gain ties in all prior waves of the network's evolution could artificially suppress the coefficient size on all ties relative to churn ties. To address this concern, here we divide the counts of ties observed in the churn period and all prior periods by the number of waves in the period. Because the

churn period contains six waves of tie formation and the all prior periods contain $t-1$ waves, we divide the tie counts by six and $t-1$, respectively. This frequency-weighted analysis addresses the natural question of whether the above results are simply an artifact of the larger number of opportunities to gain edge weights in all $t-1$ waves. Table S4 displays the results of this frequency-weighted analysis. The results indicate that ties formed during the churn period alone have a log-odds coefficient of 3.22. All ties observed in the periods that precede a given wave of the interaction network have a coefficient of 5.49. Thus, although the coefficient on all ties is larger than the coefficient on churn ties ($z = 6.03$), it is noteworthy that each churn tie explains $3.22/5.49 = 59\%$ of variance in subsequent interaction tie formation. That is, ties observed in only the first six days of the program in turn explain the majority of variance in tie evolution.

Figure S5 displays the results of cross-sectional ERGMs fitted to each period that use only network density and churn ties as predictors. The presence of a tie during the churn period associates consistently with an increase in the odds of a tie in later periods. Although the odds decrease slightly over time, two individuals who met during the churn phase enjoy a nearly 6 times greater chance of reporting a tie during the final days of the survey. On average, two nodes who met each other during the churn phase are 5 times more likely to report a tie in subsequent periods, with a maximum increase in the odds of 8 and a minimum of 2. Each coefficient is significant at the $\alpha = .05$ level. These results suggest that the effect of a churn tie is relatively durable over time. Cross-sectional ERGM analyses were conducted using the 'ergm' package (6, 7) in the R statistical computing environment (2).

Further, while the above results suggest that the earliest periods of network formation provide explanatory information about the interaction network's subsequent evolution, here we examine the extent to which those effects extend to the evolution of the scientific collaboration network, namely whether the earliest interaction ties explain disproportionate variance in scientific collaboration ties. To accomplish this task, we compare a model specified with only churn ties to a model specified with all prior

Table S4: TERGMs: Evolution of the Interaction Network (churn vs. all ties, frequency weighted)

	**Model 1**	**Model 2**
Edges	-4.99^*	-5.49^*
	[−5.15; −4.86]	[−5.72; −5.31]
Churn Ties (Freq.)	3.22^*	
	[2.90; 3.50]	
All Previous Ties (Freq.)		5.49^*
		[4.91; 6.25]
In-stars (2)	0.11^*	0.10^*
	[0.07; 0.15]	[0.05; 0.14]
Out-stars (2)	0.14^*	0.14^*
	[0.13; 0.15]	[0.13; 0.15]
4-cycles	-0.34^*	-0.35^*
	[−0.52; −0.20]	[−0.54; −0.21]
GWESP (fixed = 0)	0.38^*	0.36^*
	[0.28; 0.49]	[0.26; 0.48]
Homophily (Gender)	−0.00	0.00
	[−0.11; 0.10]	[−0.11; 0.11]
Homophily (Nationality)	0.06	0.07
	[−0.01; 0.13]	[−0.01; 0.14]
Homophily (Ethnicity)	0.04	0.02
	[−0.07; 0.15]	[−0.08; 0.14]
Memory (Stability)	0.86^*	0.44^*
	[0.76; 0.97]	[0.33; 0.56]

Note: A star indicates significance at the $\alpha = .05$ level. Ties are weighted by frequency (i.e. counts) of daily interactions.

interactions that precede a given wave of the scientific network, weighting ties by frequency (i.e. counts) of daily interactions. Table S5 displays the results. The results suggest that ties formed during the churn period alone have a log-odds coefficient of 0.20. In contrast, all ties observed in the periods that precede a given wave of the scientific network have a coefficient of 0.11. We note that the churn tie coefficient is significantly larger than the coefficient on all ties ($z = 2.27$) according to a standard Z-test for regression coefficient equality (5). Together, these results suggest that each churn tie explains approximately $.20/.11 = 1.82$ times more variance in scientific tie evolution than a tie in any given period that precedes

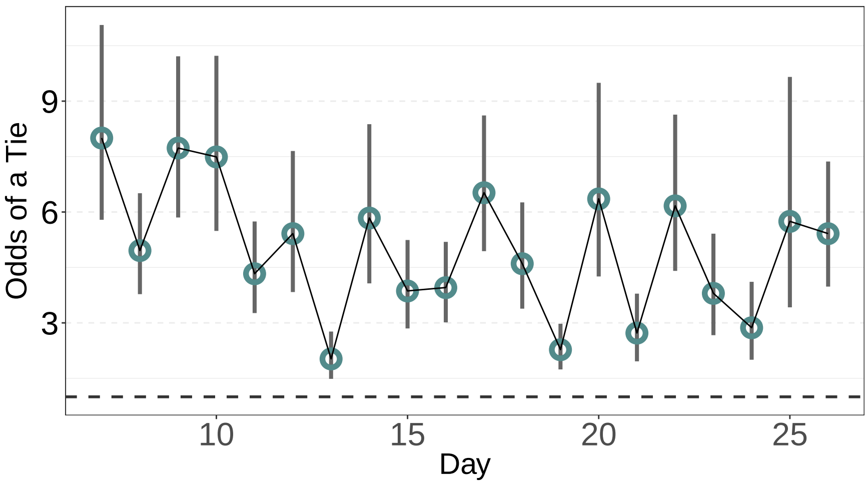

Figure S5: **Change in the odds of a tie over time given the presence of a churn tie.** Results of cross-sectional ERGMs fitted to each time slice with network density and churn ties as predictors. Although the churn ties exhibit slightly decreasing influence on tie formation over time, churn ties serve as a relatively consistent predictor of tie formation in each time slice.

a wave of the scientific network.

The above results provide initial leverage on the extent to which churn ties explain disproportionate variance in scientific tie formation. However, as in the interaction network analysis above, the results could be skewed by the fact that the number of waves in the churn period is smaller than the number of waves in all periods prior to t. Here, we divide the counts of ties observed in the churn period and all prior periods by the number of waves in the period to examine whether the above results are simply an artifact of the larger number of opportunities to gain edge weights in all $t-1$ waves. Table S6 displays the results of this frequency-weighted analysis. The results indicate that ties formed during the churn period alone have a log-odds coefficient of 1.20. All ties observed in the periods that precede a given wave of the interaction network have a coefficient of 2.22. Thus, although the coefficient on all ties is larger than the coefficient on churn ties ($z = 2.61$), it is noteworthy that each churn tie explains $1.20/2.22 = 54\%$ of variance in subsequent interaction tie formation. That is, ties observed in only the first six days of the program in turn explain the majority of variance in scientific tie evolution.

Furthermore, although the daily nature of the survey provides relatively fine-grained network evo-

Table S5: TERGMs: Evolution of the Scientific Collaboration Network (churn vs. all ties)

	**Model 1**	**Model 2**
Edges	-4.56^{*}	-4.62^{*}
	[−19.17; −4.32]	[−19.20; −4.39]
Churn Ties	0.20^{*}	
	[0.18; 0.23]	
All Previous Ties		0.11^{*}
		[0.06; 0.21]
Stars (2)	-0.16^{*}	-0.16^{*}
	[−0.29; −0.11]	[−0.29; −0.11]
4-cycles	0.14^{*}	0.14^{*}
	[0.13; 0.17]	[0.13; 0.17]
GWESP (fixed = 0)	4.37^{*}	4.40^{*}
	[3.57; 18.82]	[3.60; 18.82]
Homophily (Gender)	0.07	0.08
	[−0.08; 0.54]	[−0.08; 0.55]
Homophily (Nationality)	0.19^{*}	0.20^{*}
	[0.05; 0.62]	[0.06; 0.66]
Homophily (Ethnicity)	−0.04	−0.03
	[−0.63; 0.05]	[−0.62; 0.02]
Memory (Stability)	1.58^{*}	1.56^{*}
	[0.25; 2.70]	[0.25; 2.71]

Note: A star indicates significance at the $\alpha = .05$ level. Ties are weighted by frequency (i.e. counts) of daily interactions.

lution data, the daily nature of the survey could imply that not all participants would participate every single day (e.g. due to time constraints). Thus, to ensure that the results are not highly sensitive to daily fluctuations in reporting behavior, here we aggregate these daily data into larger periods according to a moving window. We use a symmetric three-day smoothing window around t, such that we consider a tie present at time t if at least one tie was observed in the dyad at $t-1$, t, or $t+1$. This window allows us to smooth out potential "choppiness" in reporting. Table S7 reports these results. The results suggest that the churn tie effect remains large and significant. Although the size of the coefficient decreases, this is to be expected. Because the outcome network contains fewer ties after transformation, the smaller churn

Table S6: TERGMs: Evolution of the Scientific Collaboration Network (churn vs. all ties, frequency weighted)

	**Model 1**	**Model 2**
Edges	-4.56^{*}	-4.58^{*}
	[−19.17; −4.32]	[−19.20; −4.38]
Churn Ties (Freq.)	1.20^{*}	
	[1.08; 1.36]	
All Previous Ties (Freq.)		2.22^{*}
		[1.48; 3.11]
Stars (2)	-0.16^{*}	-0.16^{*}
	[−0.29; −0.11]	[−0.29; −0.11]
4-cycles	0.14^{*}	0.14^{*}
	[0.13; 0.17]	[0.13; 0.17]
GWESP (fixed = 0)	4.37^{*}	4.39^{*}
	[3.57; 18.82]	[3.60; 18.82]
Homophily (Gender)	0.07	0.08
	[−0.08; 0.54]	[−0.08; 0.55]
Homophily (Nationality)	0.19^{*}	0.19^{*}
	[0.05; 0.62]	[0.06; 0.66]
Homophily (Ethnicity)	−0.04	−0.06
	[−0.63; 0.05]	[−0.62; 0.02]
Memory (Stability)	1.58^{*}	1.59^{*}
	[0.25; 2.70]	[0.25; 2.71]

Note: A star indicates significance at the $\alpha = .05$ level. Ties are weighted by frequency (i.e. counts) of daily interactions.

coefficient simply reflects the fact that each of those outcome ties are more "valuable" (i.e. when the outcome network contains more ties, the churn effect should indeed correlate with a larger likelihood of enjoying at least one of those ties). We note that other coefficients in the model remain substantively unchanged.

Table S7: TERGMs: Evolution of the Interaction Network (3-day Smoothing Window)

	**Model 1**	**Model 2**
Edges	-5.04^*	-4.38^*
	[−5.20; −4.90]	[−4.58; −4.15]
Churn Ties	1.12^*	0.61^*
	[0.97; 1.26]	[0.44; 0.77]
In-stars (2)	0.11^*	0.09^*
	[0.07; 0.15]	[0.06; 0.11]
Out-stars (2)	0.14^*	0.10^*
	[0.13; 0.15]	[0.09; 0.11]
4-cycles	-0.33^*	-0.02^*
	[−0.51; −0.18]	[−0.03; −0.01]
GWESP (fixed = 0)	0.39^*	0.43^*
	[0.29; 0.51]	[0.33; 0.55]
Homophily (Gender)	0.01	0.05
	[−0.10; 0.12]	[−0.04; 0.14]
Homophily (Nationality)	0.12^*	0.14^*
	[0.05; 0.19]	[0.04; 0.23]
Homophily (Ethnicity)	0.06	0.05
	[−0.04; 0.17]	[−0.07; 0.17]
Memory (Stability)	0.95^*	2.17^*
	[0.85; 1.05]	[2.07; 2.29]

Note: A star indicates significance at the $\alpha = .05$ level.

As a further check on whether variation in participation influences the inferential results, and particularly the churn effect, here we incorporate an indicator variable into the interaction network model that captures whether or a not a given node participated on a given day. The inclusion of this variable controls for idiosyncrasies in day-to-day reporting. Table S8 presents the results. Model 1 presents

the original results for the churn model reported in Fig. 2(A). Model 2 presents the same model, with the addition of the participation covariate. As expected, the coefficient on participation is positive and significant. That is, participants are more likely to have a tie in the outcome network on the days that they participate in the survey, because they themselves are reporting ties. More importantly, however, the churn tie effect remains robust to inclusion of this participation covariate, which increases our confidence that the churn effect does not reduce to variations in daily participation.

Table S8: TERGMs: Evolution of the Interaction Network (Controlling for Participation)

	Model 1	Model 2
Edges	-5.04^*	-5.88^*
	[−5.20; −4.90]	[−6.07; −5.74]
Churn Ties	1.12^*	1.05^*
	[0.97; 1.26]	[0.92; 1.17]
Daily Participant		1.52^*
		[1.36; 1.73]
In-stars (2)	0.11^*	0.16^*
	[0.07; 0.15]	[0.12; 0.21]
Out-stars (2)	0.14^*	0.11^*
	[0.13; 0.15]	[0.11; 0.12]
4-cycles	-0.33^*	-0.76^*
	[−0.51; −0.18]	[−1.06; −0.59]
GWESP (fixed = 0)	0.39^*	0.18^*
	[0.29; 0.51]	[0.08; 0.29]
Homophily (Gender)	0.01	−0.00
	[−0.10; 0.12]	[−0.11; 0.10]
Homophily (Nationality)	0.12^*	0.12^*
	[0.05; 0.19]	[0.06; 0.19]
Homophily (Ethnicity)	0.06	0.04
	[−0.04; 0.17]	[−0.04; 0.14]
Memory (Stability)	0.95^*	0.90^*
	[0.85; 1.05]	[0.81; 0.99]

Note: A star indicates significance at the $\alpha = .05$ level.

A related concern is that the large variation in reported ties during week one could be skewed by

individuals who seldom participated in the survey, especially those individuals who did not participate during the later periods of the survey. Here, we subset the node set according to participation level in order to determine whether individuals who participate very seldom in the survey skew the finding that the network goes through an initial period of high levels of interactions followed by a decrease in the numbers of interactions (i.e. the churn process). Figure S6 shows the mean ties reported to alters on each day, subsetted for individuals who participated in the survey more than twice, more than six times, more than ten times, more than 14 times, and more than 18 times. In each case, the mean number of ties during the earliest periods of the network's emergence is greater than the mean number of ties during later periods of network evolution. Furthermore, Fig. S7 conducts the same procedure, except reports variance in tie counts over time. Again, we find that variance in tie counts is higher during the earliest periods of network emergence. These findings suggest that our churn concept – a period marked by a spike in average ties and high fluctuations in those ties – does not appear to reduce to individuals who participate very seldom in the survey.

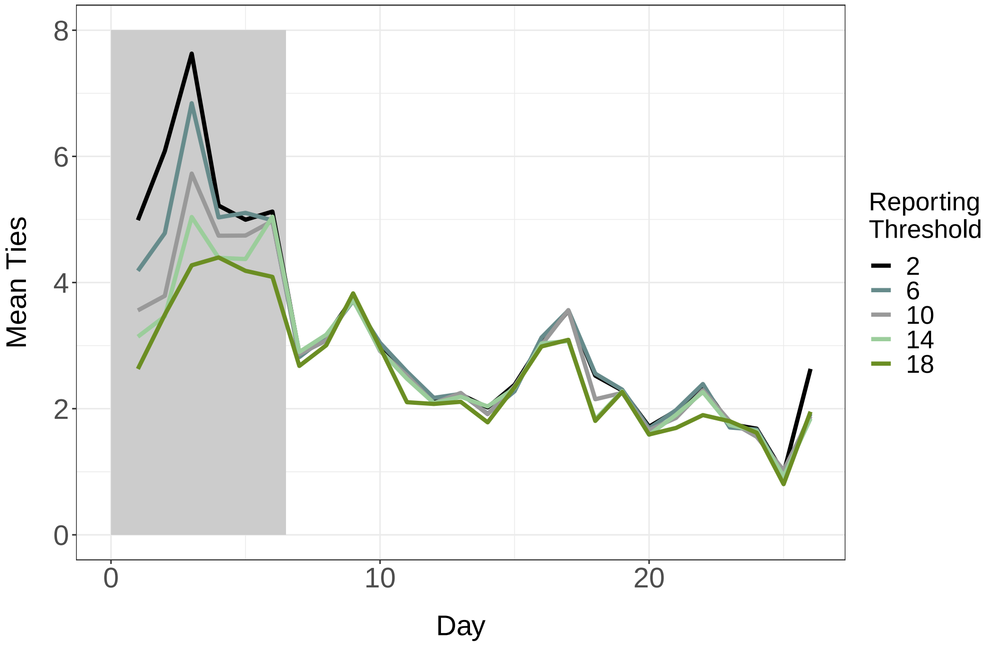

Figure S6: **Mean Ties to Alters by Participation Thresholds.**

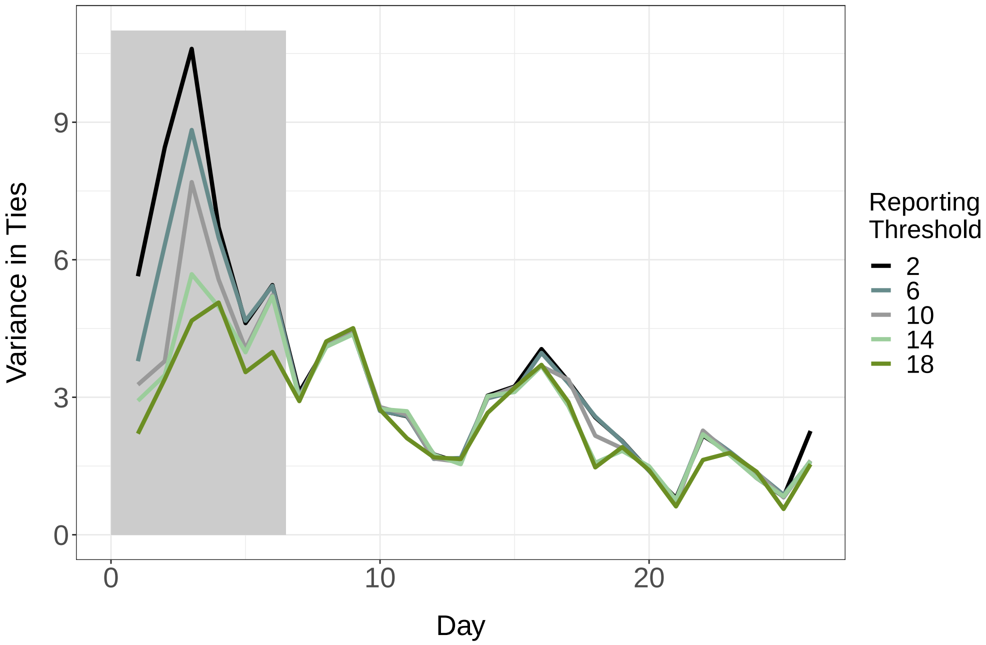

Figure S7: **Variance in Ties by Participation Thresholds.**

Finally, the daily interactions that participants report could be biased by prior interactions before the program, roommate assignment, or a number of homophily-based effects. These factors could induce endogeneity in the model, since these factors could also in turn explain later interactions and scientific collaboration patterns. Here, we examine whether the churn tie effect remains robust to the inclusion of such variables. Table S9 reports the results for the extended model of the interaction network. As expected, participants are more likely to interact with individuals with whom they share research interests and hobbies, as well as alters at similar stages of their career. Roommates and individuals who knew each other prior to the program are also more likely to interact. Importantly, however, the size and significance of the churn tie effect remains substantively unchanged. Table S10 reports the results for the extended model of the scientific collaboration network. Here, we find less evidence of homophily-induced research collaboration. We note that the training program was highly interdisciplinary, which explains the lack of significance of homophily in research interests. For example, the final co-authored papers often included a range of authors across the natural and social sciences. Alternatively, it could be

the case that the diversity of research interests in a sample of this size inhibits the recovery of homophily effects due to statistical power constraints. Importantly, the size and significance of the churn tie effect remains substantively unchanged.

Table S9: Extended TERGM: Evolution of the Interaction Network

	**Model 1**
Edges	-5.42^{*}
	$[-5.61; -5.24]$
Churn Ties	1.05^{*}
	$[0.91; 1.20]$
In-stars (2)	0.13^{*}
	$[0.09; 0.17]$
Out-stars (2)	0.15^{*}
	$[0.14; 0.15]$
4-cycles	-0.53^{*}
	$[-0.76; -0.38]$
GWESP (fixed = 0)	0.33^{*}
	$[0.23; 0.44]$
Reciprocity	1.55^{*}
	$[1.39; 1.69]$
Homophily (Gender)	-0.00
	$[-0.11; 0.11]$
Homophily (Nationality)	0.09^{*}
	$[0.01; 0.17]$
Homophily (Ethnicity)	0.08
	$[-0.02; 0.20]$
Homophily (Research interests)	0.21^{*}
	$[0.13; 0.29]$
Homophily (Career stage)	0.26^{*}
	$[0.16; 0.36]$
Homophily (Hobbies)	0.19^{*}
	$[0.05; 0.33]$
Roommates	1.20^{*}
	$[0.79; 1.51]$
Contact Prior to Program	0.88^{*}
	$[0.55; 1.17]$
Memory (Stablity)	0.93^{*}
	$[0.83; 1.02]$

Note: A star indicates significance at the $\alpha = .05$ level.

Table S10: Extended TERGM: Evolution of the Scientific Collaboration Network

	**Model 1**
Edges	-4.56^{*}
	$[-30.12; -4.33]$
Churn Ties	0.33^{*}
	$[0.11; 0.47]$
Stars (2)	-0.16^{*}
	$[-0.29; -0.11]$
4-cycles	0.14^{*}
	$[0.14; 0.18]$
GWESP (fixed = 0)	4.40^{*}
	$[3.54; 29.89]$
Homophily (Gender)	0.10
	$[-0.16; 0.68]$
Homophily (Nationality)	0.08
	$[-0.12; 0.71]$
Homophily (Ethnicity)	-0.05
	$[-0.57; 0.14]$
Homophily (Research interests)	0.20
	$[-0.05; 0.80]$
Homophily (Career stage)	0.08
	$[-0.28; 0.43]$
Homophily (Hobbies)	-0.34^{*}
	$[-0.44; -0.19]$
Roommates	-0.05
	$[-13.64; 1.78]$
Contact Prior to Program	1.22^{*}
	$[1.09; 1.67]$
Memory (Stability)	1.59^{*}
	$[0.27; 2.80]$

Note: A star indicates significance at the $\alpha = .05$ level.

Goodness-of-Fit Diagnostics

To assess the adequacy of the fit of the interaction network TERGMs, we use our fitted models to simulate 100 networks for each time slice and compare the simulations of the model with churn ties specified against the model with no churn ties. Figure S8 displays the results of this exercise, with box plots representing the simulated values and the black lines representing the observed values for the statistics of dyad-wise shared partners and indegree. The bottom row of the figure displays the sums of the areas under the receiver operating characteristic (ROC) curves for each time slice. Both models display relatively similar fits, with modest improvement in the average area under the ROC curve ($\bar{x}_1 = 0.75$ vs $\bar{x}_2 = 0.70$, $p = 0.01$, $t = 2.32$). Furthermore, we note that the goodness-of-fit results improve when coercing 3 day periods of the outcome network into one observed network slice (akin to a smoothing window). This improvement increases our confidence that any lack of in-sample fit reported here is more attributable to dependence on participation in a given day rather than variables omitted from the model itself.

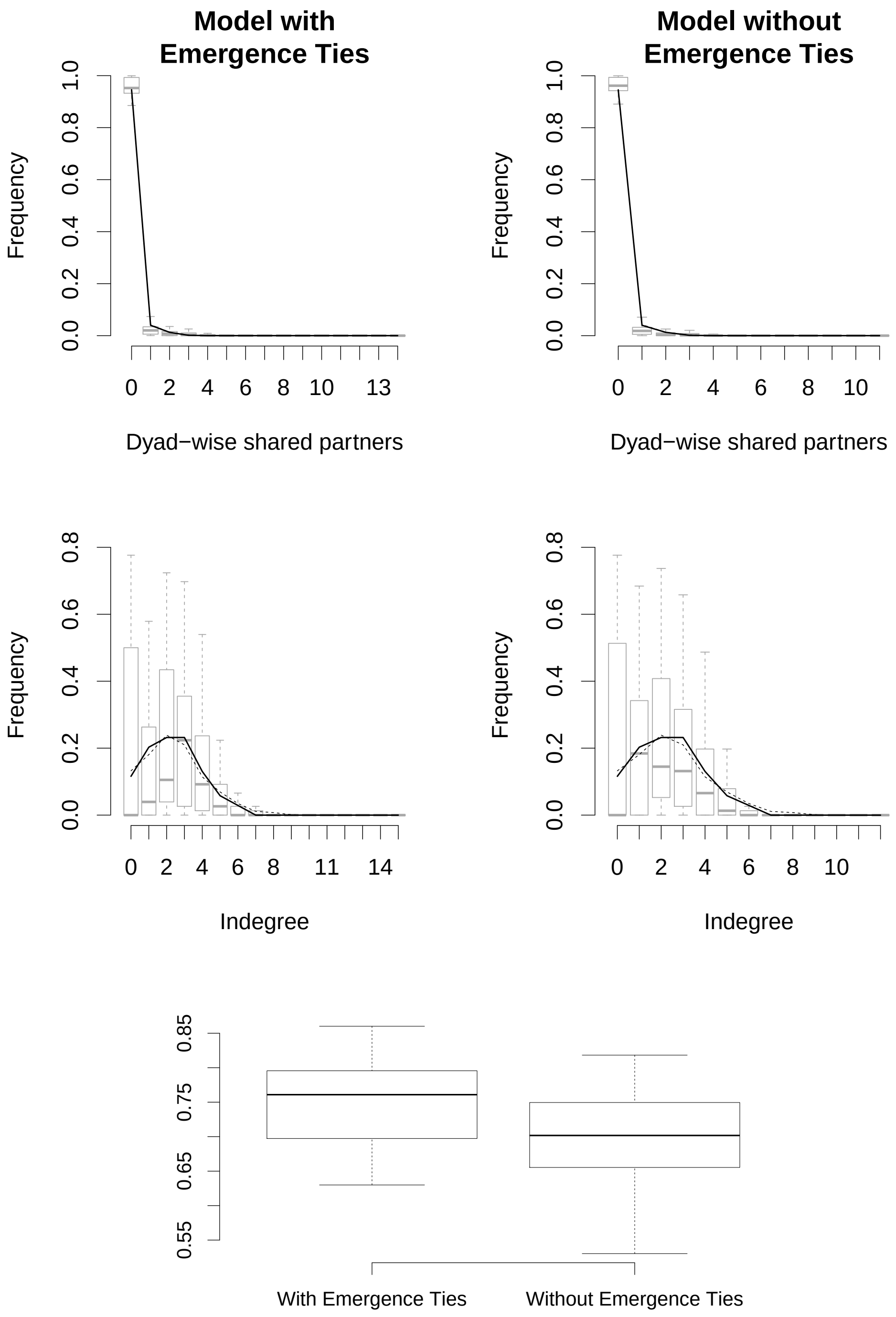

Figure S8: **In-Sample Goodness-of-Fit.** In-sample goodness-of-fit diagnostics for the interaction network model with churn ties and the model without churn ties based on 100 simulated networks at each time slice. In the top 2 rows, box plots represent the simulated statistics and black lines represent the observed statistic values. In the bottom row, box plots represent sums of the areas under the ROC curve calculated at each time slice.

Additional Model Simulations

In the main paper, we report model simulation results for the parameter settings $\alpha = 0.45$, $p_{\text{gain}} = 0.07$, and $b = 0.21$. To obtain these parameter values, we simulate densities from all reasonable combinations of parameters and then select the set of parameters that minimize the sum of squared error between the simulated and observed density plots. Figure S9(A) plots the observed and simulated levels of density, tie innovation, and tie loss for this set of parameters. Furthermore, in Figure S9(B) we illustrate the ways in which simulated levels of density change due to adjustments in the sociality, bandwidth, and time cost parameters. As would be expected, higher levels of sociality, higher social bandwidths, and lower time costs produce networks with higher tie counts, all else equal.

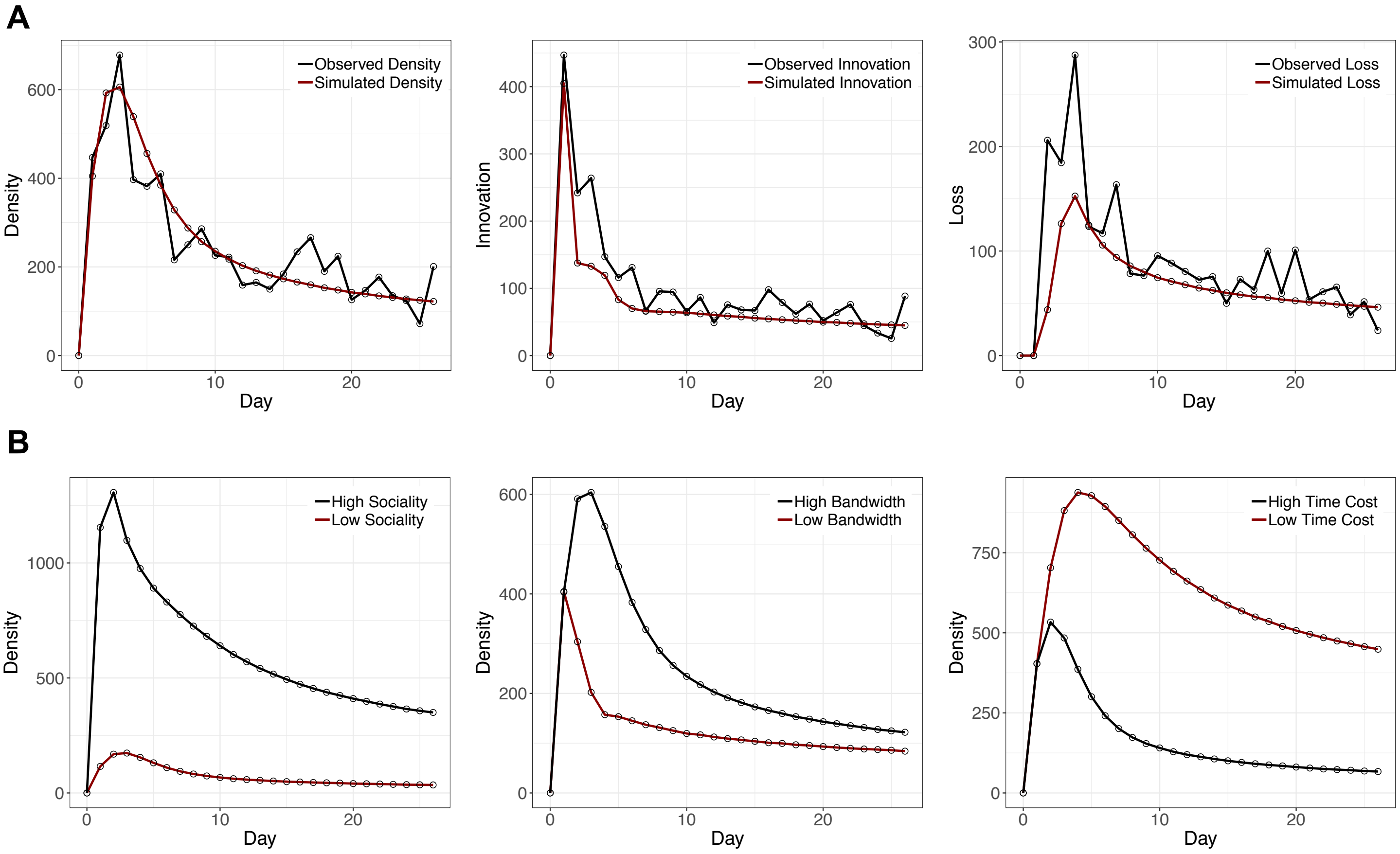

Figure S9: **Additional model simulations.** In (A), the mean results from 1,000 simulation runs for the parameters reported in the main paper for density (i.e. counts of ties in the network), innovation (i.e. counts of ties observed in period t that did not exist at $t-1$, and loss (i.e. counts of ties that existed in $t-1$ that no longer exist in period t). In (B), illustrations of how the mean simulated density changes due to the adjustment of the model's sociality, bandwidth, and time cost parameters while holding other parameters constant. All else equal, higher levels of sociality, higher social bandwidths, and lower time costs produce networks with higher tie counts. Confidence intervals become too narrow to visualize as the number of simulations increases. Thus, these trend lines represent the statistic counts to which the model converges.

References

[1] H. Wickham, *ggplot2: Elegant Graphics for Data Analysis*. Springer-Verlag New York, 2016.

[2] R Core Team, *R: A Language and Environment for Statistical Computing*. R Foundation for Statistical Computing, Vienna, Austria, 2018.

[3] M. Á. Serrano, M. Boguná, and A. Vespignani, "Extracting the multiscale backbone of complex weighted networks," *Proceedings of the National Academy of Sciences*, vol. 106, no. 16, pp. 6483–6488, 2009.

[4] P. Leifeld, S. J. Cranmer, and B. A. Desmarais, "Temporal exponential random graph models with btergm: Estimation and bootstrap confidence intervals," *Journal of Statistical Software*, vol. 83, no. 6, 2018.

[5] R. Paternoster, R. Brame, P. Mazerolle, and A. Piquero, "Using the correct statistical test for the equality of regression coefficients," *Criminology*, vol. 36, no. 4, pp. 859–866, 1998.

[6] M. S. Handcock, D. R. Hunter, C. T. Butts, S. M. Goodreau, P. N. Krivitsky, and M. Morris, *ergm: Fit, Simulate and Diagnose Exponential-Family Models for Networks*. The Statnet Project (http://www.statnet.org), 2018. R package version 3.9.4.

[7] D. R. Hunter, M. S. Handcock, C. T. Butts, S. M. Goodreau, and M. Morris, "ergm: A package to fit, simulate and diagnose exponential-family models for networks," *Journal of Statistical Software*, vol. 24, no. 3, pp. 1–29, 2008.
